# Steady-State Visual Evoked Potential Classification Using Complex Valued Convolutional Neural Networks

**DOI:** 10.3390/s21165309

**Published:** 2021-08-06

**Authors:** Akira Ikeda, Yoshikazu Washizawa

**Affiliations:** Department of Computer and Network Engineering, The University of Electro-Communications, Tokyo 182-8585, Japan; channike500@gmail.com

**Keywords:** brain–computer interfaces (BCI), steady-state visual evoked potential (SSVEP), complex valued deep neural networks

## Abstract

The steady-state visual evoked potential (SSVEP), which is a kind of event-related potential in electroencephalograms (EEGs), has been applied to brain–computer interfaces (BCIs). SSVEP-based BCIs currently perform the best in terms of information transfer rate (ITR) among various BCI implementation methods. Canonical component analysis (CCA) or spectrum estimation, such as the Fourier transform, and their extensions have been used to extract features of SSVEPs. However, these signal extraction methods have a limitation in the available stimulation frequency; thus, the number of commands is limited. In this paper, we propose a complex valued convolutional neural network (CVCNN) to overcome the limitation of SSVEP-based BCIs. The experimental results demonstrate that the proposed method overcomes the limitation of the stimulation frequency, and it outperforms conventional SSVEP feature extraction methods.

## 1. Introduction

The brain–computer interface (BCI) or brain–machine interface (BMI) is a direct communication pathway for controlling external devices by discriminating brain signals [1,2]. BCIs have been widely researched and applied for many practical systems [3,4,5,6]. There are various methods for acquiring brain signals for BCIs such as electroencephalography (EEG), magnetoencephalography (MEG), and functional magnetic resonance imaging (fMRI). In this paper, we focus on EEG-based BCI because EEG is a noninvasive and simple signal acquisition method for BCIs [7].

There are various ways to issue commands for BCIs. For example, event-related potentials (ERPs) such as P300 [8], sensorimotor rhythms (SMRs) such as μ-rhythm [9,10], the steady-state visual evoked potential (SSVEP), and auditory or tactile evoked responses [11,12] have been used for BCIs. A recent review of BCI technologies and applications appeared in [13].

SSVEP is a response to periodic flicker stimulation with a frequency of 5–30 Hz that is usually presented by an LED or LCD [14,15]. The response EEG has peak frequencies that are the fundamental and harmonic frequencies of the flicker stimulation. An SSVEP-based BCI is realized by assigning commands or alphabets to flickers having different frequencies [16,17]. BCIs can determine the intended commands of users by detecting the peak frequency from observed EEGs. SSVEP-based BCIs achieve the highest information transfer rate (ITR) among state-of-the-art BCI implementations [16,17,18]. There are many extensions and hybrid BCIs for SSVEP-based BCIs. For example, [19,20] extended the SSVEP-based BCI by utilizing the phase of the stimulus in addition to frequency information.

The command of SSVEP-based BCIs is determined by a frequency analysis of the observed EEGs. In the early days, Fourier analysis or spectrum estimation methods were used [21,22]. Then, canonical component analysis (CCA)-based approaches were developed [23]. There are various extensions for CCA-based methods that determine the command of SSVEP-based BCIs. Multi-way CCA (mwayCCA) was developed for tensor EEG data [24]. Phase-constrained CCA (p-CCA) was proposed for phase-modulated SSVEP BCIs [25]. Filter bank canonical correlation analysis (FBCCA) decomposes EEGs by using a sub-band filter bank and then applies CCA to each sub-band signal [26]. The individual template-based CCA (IT-CCA) integrates the standard CCA and the canonical correlation between individual templates and test data [27].

Although the SSVEP-based BCI achieves performances well among state-of-the-art BCI implementations, it and its signal processing methods still have drawbacks to be addressed. LCD is often used to present SSVEP stimuli, and it normally has a fixed refresh rate of 60 Hz. Thus, the stimulus frequency is basically limited to a sub-multiple of the refresh rate. Although an approximation method for generating a stimulus in which the frequency is not sub-multiples of the monitor refresh rate was proposed [28], it is not an essential solution and it requires a longer analysis time window. Another problem is due to Fourier analysis or CCA-based feature extraction. They extract not only the stimulus flicker frequency but also its harmonic components. Therefore, these methods cannot discriminate two flicker frequencies when one frequency is twice (or three times) that of the other, and these stimulus frequency pairs are not available for current SSVEP-based BCIs. These two problems prevent an increase in the number of BCI commands and ITR.

To address these problems, we propose a complex valued convolutional neural network (CVCNN) for SSVEP classification. To the best of our knowledge, this paper is the first to apply CVCNN to an SSVEP classification problem, and it proves that CVCNN overcomes the problem of stimulus frequency limitation. The combination of feature extraction using the discrete Fourier transform (DFT) and convolutional neural networks (CNNs) has been applied to SSVEP classification and has shown higher ITR performance [29]. However, the biggest advantage of CNNs is the data-driven automatic learning of optimal feature extraction, called representation learning [30]. Feature extraction using DFT may not be optimal for SSVEP data, and the combination of DFT and CNN cannot extract optimal features from EEG data directly. Any DFT coefficient is represented by the complex inner product between an input signal and the discrete Fourier basis; hence, we propose using a complex valued neural networks for feature extraction. DFT is a fixed basis for representing an input signal; on the other hand, a complex weight vector of a neural network is a variable parameter that is optimized with respect to an appropriate criterion represented by a cost function or loss function. In other words, by virtue of complex valued convolutional neural networks and representation learning, the network obtains the optimal feature extraction including DFT. We introduce the activation function using a complex absolute value function that is equivalent to calculating the amplitude spectrum for a fixed complex Fourier basis [31].

We conducted an SSVEP-based BCI experiment in order to demonstrate the performance of the proposed CNN and compared it with conventional methods. In our experiment, the flicker stimulations had pair flickers in which one frequency was twice that of the other. We also show the experimental results obtained using open SSVEP-based BCI data. The experiments demonstrate that the proposed method can discriminate pair commands, and it outperforms the conventional methods for SSVEP-based BCIs.

The rest of the paper is organized as follows. Section 2 explains complex valued neural networks and their learning procedure. Section 3 describes the experimental setting of our SSVEP-based BCI and the open dataset. Section 4 shows the experimental results, comparing the proposed method with conventional methods. Section 5 concludes the paper.

## 2. Method: Complex Valued Neural Networks (CVCNN)

### 2.1. Activation Function for CVCNN

Complex feature vectors represent periodic/cyclic data, such as oscillation and wave, e.g., electromagnetic phenomena, electric circuits, acoustic/biomedical signals, and imaging radar, in a natural way. Complex valued neural networks (CVNN) have been used for array signal analysis [32,33], radar image processing [34,35], and EEG analysis [36,37,38].

Unlike ordinary real valued neural networks (RVNNs), the activation function of CVNNs has to be considered carefully because complex functions have a restriction in terms of differentiability expressed by Cauchy–Riemann equations. The activation functions of CVNNs are categorized into two groups: the split type $f\left(z\right)=\phi_{1}\left(ℜ\left(z\right)\right)+j\phi_{1}\left(ℑ\left(z\right)\right)$ and the amplitude-phase type $f\left(z\right)=\phi_{2}\left(|z|\right)\exp\left(j\arg z\right)$, where $j=\sqrt{-1}$ is an imaginary unit, and ℜ(z) and ℑ(z) are real and imaginary parts of z∈C, respectively. For the split type, the rectified linear unit (ReLU) function $\phi_{1}\left(x\right)=\max\left(x,0\right)$ or the hyperbolic tangent function $\phi_{1}\left(x\right)=\tanh\left(x\right)$ is often used. For the amplitude-phase type, $\phi_{2}\left(x\right)=\tanh\left(x\right)$ is used [39,40]. A review of CVNN and of their applications in signal processing can be found in [41].

**Figure 1:**
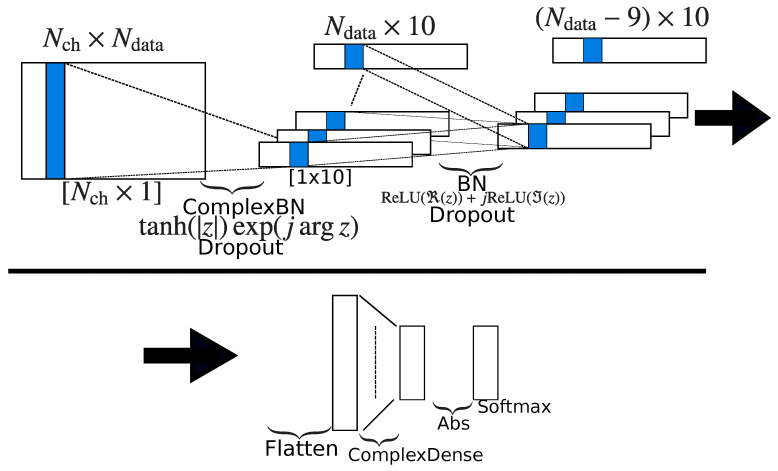
Network structure of the proposed CNN: the first layer is the complex weighted averaging for channels (2–4 in Table 1). The second layer is the filtering for time index (5–8 in Table 1). Then, the data are flattened and goes through the complex dense layer with the absolute activation function. Finally, the output is given by the softmax layer.

Phase information in EEGs or other signals is often unnecessary because it depends on the onset of data. Some SSVEP-based BCIs utilize absolute or relative phase information to increase the number of commands. However, in these methods, the BCI system should carefully synchronize EEG recording and stimulus presentation. Here, we consider discrimination of the stimulus frequency; therefore, phase information should be removed. For this purpose, the authors previously proposed the use of the complex absolute function ϕ(z)=|z| for the activation function [31]. In the proposed method, we use three activation functions: the amplitude-phase and split types, and the absolute function. The structure of the proposed neural networks is shown in Table 1 and Figure 1. The complex version of batch normalization proposed in [40] is used.

### 2.2. Forward and Backward Propagation

Let $W^{\left(l\right)}$ be the weight matrix and $o^{\left(l\right)}$ be the output vector of the *l*th layer of the network. Then, the forward propagation of an *L* layer CVCNN is calculated by the following equations:

(1) $$\begin{array}{cc} u^{\left(l\right)}= & W^{\left(l\right)}o^{\left(l-1\right)} \end{array}$$

(2) $$\begin{array}{cc} o^{\left(l\right)}= & \phi^{\left(l\right)}\left(u^{\left(l\right)}\right),\;l=2,\ldots ,L, \end{array}$$

where $\phi^{\left(l\right)}$ is the element-wise activation function of the *l*th layer and $o^{\left(1\right)}$ denotes the complex valued input vector of the network.

The set of weight matrices $W={\left\{W^{\left(l\right)}\right\}}_{l=1,\ldots ,L}$ is optimized to minimize a loss function by back-propagation (BP) for CVCNNs. Square-error loss, logistic loss, or softmax loss is often used for classification problems. Let $E_{n}\left(W\right)$ be the loss function for the *n*th input training sample. Each weight matrix is iteratively updated by

(3) $$\begin{array}{c} W^{\left(l\right)}\leftarrow W^{\left(l\right)}-\eta \nabla_{W^{\left(l\right)}}E_{n}\;l=2,\ldots ,L, \end{array}$$

where η>0 is the learning rate.

#### 2.2.1. Split-Type Activation Function

Suppose that we use a split-type activation function. Let $u_{r}^{\left(l\right)}$ be the *r*th element of $u^{\left(l\right)}$, i.e., $u^{\left(l\right)}={\left[u_{1}^{\left(l\right)},u_{2}^{\left(l\right)},\ldots ,u_{m}^{\left(l\right)}\right]}^{⊤}$, $w_{rp}^{\left(l\right)}$ is the (r,p) element of $W^{\left(l\right)}$, and

(4) $$\begin{array}{c} \delta_{r}^{\left(l\right)}=\frac{\partial E}{\partial ℜ[u_{r}^{\left(l\right)}]}+j\frac{\partial E}{\partial ℑ[u_{r}^{\left(l\right)}]}. \end{array}$$

Then, applying the chain rule for real and imaginary parts independently, we have

(5) $$\begin{array}{cc} \frac{\partial E}{\partial w_{rp}^{\left(l\right)}}= & \frac{\partial E}{\partial ℜ[w_{rp}^{\left(l\right)}]}+j\frac{\partial E}{\partial ℑ[w_{rp}^{\left(l\right)}]}=\delta_{r}^{\left(l\right)}\bar{o_{p}^{\left(l-1\right)}},\;l=2,\ldots ,L, \end{array}$$

where $\bar{z}$ denotes the complex conjugate of *z* and $o_{p}^{\left(l-1\right)}$ is the *p*th element of $o^{l-1}$. For simplification purposes, we omit the subscript of the training index, *n*. Then, $\delta_{r}^{\left(l\right)}$ is calculated by the chain rule:

(6) $$\begin{array}{cc} ℜ[\delta_{r}^{\left(l\right)}]= & ℜ\left[\sum_{q}\delta_{q}^{\left(l+1\right)}\bar{w_{qr}^{\left(l+1\right)}}\right]\frac{\partial ℜ[\phi^{\left(l\right)}\left(u_{r}^{\left(l\right)}\right)]}{\partial ℜ[u_{r}^{\left(l\right)}]} \end{array}$$

(7) $$\begin{array}{cc} ℑ[\delta_{r}^{\left(l\right)}]= & ℑ\left[\sum_{q}\delta_{q}^{\left(l+1\right)}\bar{w_{qr}^{\left(l+1\right)}}\right]\frac{\partial ℑ[\phi^{\left(l\right)}\left(u_{r}^{\left(l\right)}\right)]}{\partial ℑ[u_{r}^{\left(l\right)}]}. \end{array}$$

#### 2.2.2. Amplitude-Phase Type

In the case of the amplitude-phase type activation function f(z)=tanh(|z|)exp(jargz), the absolute value and phase of the entries of $W^{\left(l\right)}$ are independently updated by the following rule [42,43]:

(8) $$\begin{array}{cc} |w_{rp}^{\left(l\right)}|\leftarrow & |w_{rp}^{\left(l\right)}\left|-\eta \delta \right|w_{rp}^{\left(l\right)}|,\;\arg w_{rp}^{\left(l\right)}\leftarrow \arg w_{rp}^{\left(l\right)}-\eta \delta \arg w_{rp}^{\left(l\right)},\\ \delta |w_{rp}^{\left(l\right)}|= & \left(1-|{\left(o_{r}^{\left(l\right)}\right)}^{2}|\right)\left(|o_{r}^{\left(l\right)}\left|-\right|d_{r}^{\left(l\right)}|\cos\left(\arg o_{r}^{\left(l\right)}-\arg d_{r}^{\left(l\right)}\right)\right)\left|o_{p}^{\left(l-1\right)}\right|\cos\theta_{rp}^{\left(l\right)} \end{array}$$

(9) $$\begin{array}{cc} & -|o_{r}^{\left(l\right)}\left|\right|d_{r}^{\left(l\right)}|\sin\left(\arg o_{r}^{\left(l\right)}-\arg d_{r}^{\left(l\right)}\right)\frac{o_{p}^{l-1}}{u_{r}^{\left(l\right)}}\sin\theta_{rp}^{\left(l\right)},\\ \delta \arg w_{rp}^{\left(l\right)}= & \left(1-|{\left(o_{r}^{\left(l\right)}\right)}^{2}|\right)\left(|o_{r}^{\left(l\right)}\left|-\right|d_{r}^{\left(l\right)}|\cos\left(\arg o_{r}^{\left(l\right)}-\arg d_{r}^{\left(l\right)}\right)\right)\left|o_{p}^{\left(l-1\right)}\right|\sin\theta_{rp}^{\left(l\right)} \end{array}$$

(10) $$\begin{array}{cc} & +|o_{r}^{\left(l\right)}\left|\right|d_{r}^{\left(l\right)}|\sin\left(\arg o_{r}^{\left(l\right)}-\arg d_{r}^{\left(l\right)}\right)\frac{o_{p}^{l-1}}{u_{r}^{\left(l\right)}}\cos\theta_{rp}^{\left(l\right)}, \end{array}$$

where $\theta_{rp}^{\left(l\right)}=\arg o_{r}^{\left(l\right)}-\arg o_{p}^{\left(l\right)}-\arg w_{rp}^{\left(l\right)}$, and $d^{\left(l-1\right)}=\bar{\phi^{\left(l\right)}\left(\bar{d^{\left(l\right)}}W^{\left(l\right)}\right)}$.

#### 2.2.3. Absolute Activation Function

Since the output of the absolute activation function ϕ(z)=|z| is real-valued, the layers after the absolute activation function are treated as ordinary RVNNs. Suppose that the activation function of the *l*th layer is the absolute function; then, the chain rule is given by Equations (6) and (7).

## 3. Experimental Setting and Dataset

We used two datasets: (i) our original SSVEP dataset (original dataset) and (ii) an open SSVEP benchmark dataset (open dataset) [44]. The original dataset was designed to demonstrate the distinctiveness of harmonic stimulus flicker frequencies. The stimulus flicker frequencies were 6.0, 6.5, 7.0 7.5, and 8.0 Hz, and their second harmonics were 12, 13, 14, 15, and 16 Hz. With CCA-based methods, it is difficult to classify these harmonic pairs.

### 3.1. Original Dataset

We used a refresh rate of 60 Hz, a 27-inch LCD monitor, and Psychtoolbox-3 for stimulus presentation [45]. For EEG recording, we used the MATLAB data acquisition toolbox, g.tec active EEG (g.GAMMAcap2, g.GAMMAbox, and g.LADYbird), a TEAC BA1008 amplifier, and a Contec AI-1664 LAX-USB A/D converter. The sampling frequency was 512 Hz. A reference electrode was placed on the right earlobe, and the ground electrode was placed on FPz. We used nine electrodes for recording: O1, O2, Oz, P3, P4, Pz, PO3, PO4, and POz.

Five healthy subjects (four males and one female, 26.8 ± 7.43 y.o.) voluntarily participated in the experiment. The experiment procedure was approved by the ethics committee of the University of Electro-Communications, and it was conducted in accordance with the approved research procedure and with the relevant guidelines and regulations. Informed consent was obtained from all the subjects.

**Figure 2:**
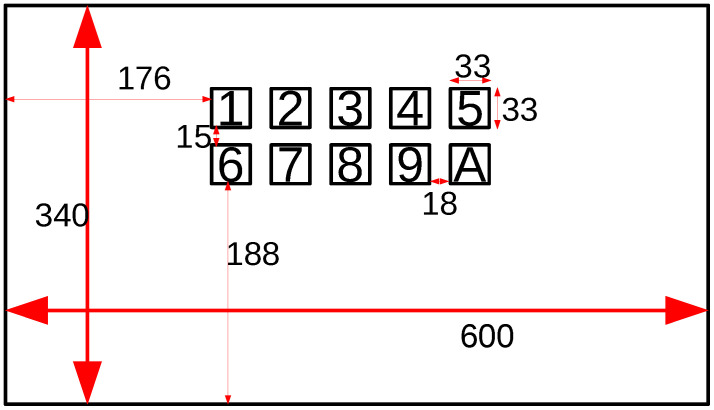
Stimulus presentation: unit, mm; 1: 6.0 Hz, 2: 6.5 Hz, 3: 7.0 Hz, 4: 7.5 Hz, 5: 8.0 Hz, 6: 15 Hz, 7: 16 Hz, 8: 12 Hz, 9: 13 Hz, and A: 14 Hz.

Figure 2 depicts the stimuli presentation setting of our experiment. The distance from the eyes of the subject to the monitor was 70–80 cm. The target queue was randomly displayed for two seconds, and flicker stimuli were then presented for five seconds. The stimuli were given with a white and black box. One trial consisted of ten randomly ordered target stimuli, and ten trials were conducted for each subject. The subjects took a rest between trials as needed. The subjects were asked not to blink during the stimulus presentation. Due to the latency of the SSVEP, we extracted EEG from 0.14 s after the start of stimulation. The length of the EEG data for one target was five seconds. We rearranged the data and generated data 1000 ms and 500 ms in length with no overlaps. For each subject, the number of pieces of data were 500 and 1200 for data of 1000 ms and 500 ms in length, respectively. We applied band-pass filtering from 4 Hz to 45 Hz. The classification accuracy and ITR were evaluated using ten-fold cross validation.

### 3.2. Open Dataset

The open dataset consisted of ten subjects’ SSVEP data [44]. There were twelve targets (9.25, 9.75, …, 14.25, and 14.75 Hz). Four-class phase modulation was also used (0π, 0.5π, 1.0π, and 1.5π). There were 15 trials for each target, and 1 trial lasted four seconds.

To utilize the phase modulation, we generated training and test data as follows: (i) applied band-pass filtering; (ii) extracted the data with offset $t_{\mathrm{off}}$ from the onset of the stimulus for each trial, with the data length being 500 ms or 1000 ms; (iii) generated training/test data by five-fold cross-validation, i.e., for each target, 12 trials for training data and 3 trials for test data; and (iv) calculated classification accuracy and ITR and averaged them for several offset values t. For the 500 ms data, we used $t_{\mathrm{off}}\in \left\{0,500,\ldots ,3500\right\}$, and for the 1000 ms data, we used $t_{\mathrm{off}}\in \left\{0,1000,\ldots ,3000\right\}$.

### 3.3. Methods for Comparison

We compared the proposed method with (i) CCA [23], (ii) combined CCA [44], and (iii) CCNN [29].

For the CCA and combined CCA, we used the fundamental and second harmonics of the stimulus flicker frequency for the reference signal. We implemented them using Python 3.6.8, Numpy 1.19.1, and Scikit-learn 0.23.1.

For the CCNN, we used the same network structure as in [29], and we applied DFT with zero-padding; then, a complex spectrum from 3 Hz to 35 Hz was input to the network in a similar manner as [29]. The frequency resolution was set to 0.293 Hz. For the CCNN and CVCNN (proposed), the network parameters were fixed or determined by the grid search listed in Table 2 and implemented by using TensorFlow 1.14.0 and Keras 2.2.4 in addition to the software above.

## 4. Results

The classification performance of the SSVEP-based BCI was evaluated in terms of the classification accuracy and ITR by using the cross validation for each subject. ITR *I* (bit/min) was obtained by the following equation:

(11) $$\begin{array}{c} I=\frac{60}{T}\left(\log_{2}M+P\log_{2}P+\left(1-P\right)\log_{2}\left(\frac{1-P}{M-1}\right)\right), \end{array}$$

where *T* (s) is the time to output the command, *P* is the mean classification accuracy, and *M* is the number of targets [2]. We assumed that the latency of the subject’s attention to the target stimulus was 0.5 s and obtained the ITR.

### 4.1. Original Dataset

**Figure 3:**
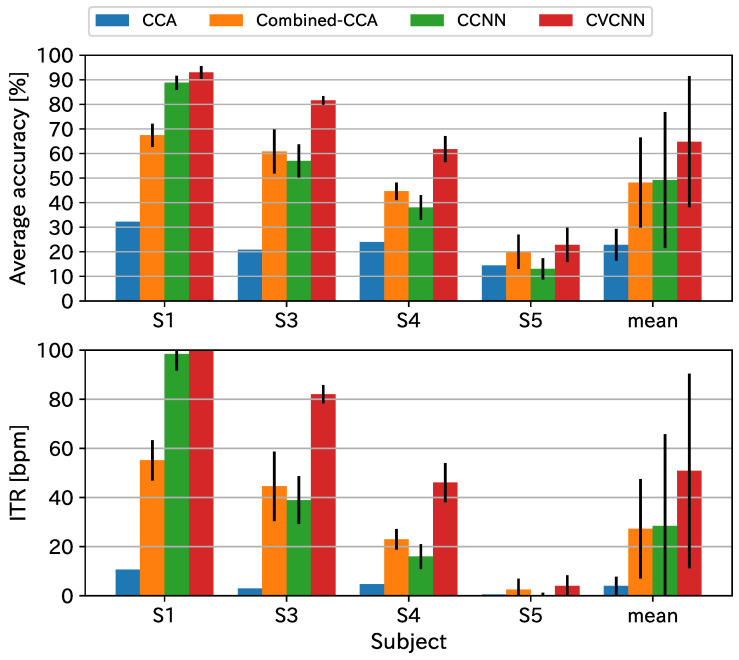
Classification accuracy and ITR for the original dataset with a data length of 1000 ms.

**Figure 4:**
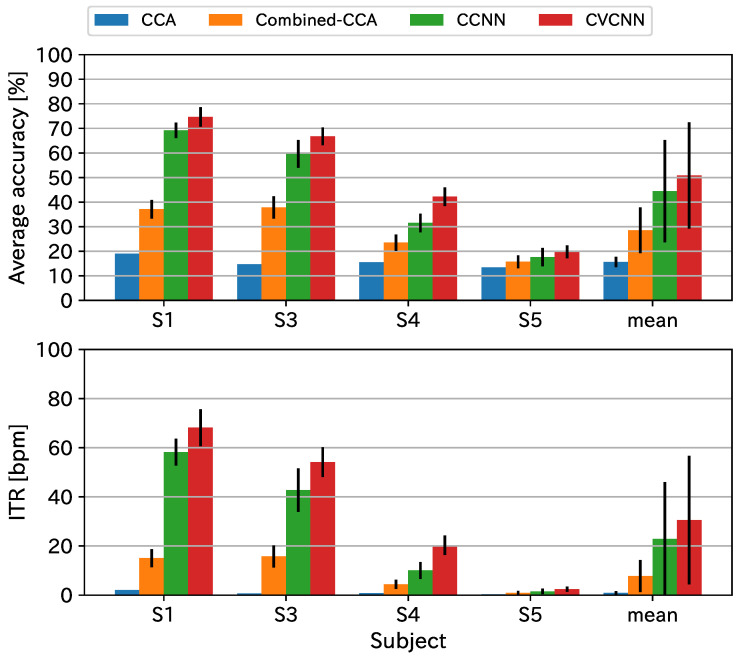
Classification accuracy and ITR for the original dataset with a data length of 500 ms.

We excluded subject 2 from the analysis because the recorded EEG data were inappropriate because some electrodes were not set up properly. Figure 3 and Figure 4 show the classification accuracies and ITR. The proposed method exhibited the best classification accuracy and ITR for all data lengths. Since the stimulus flicker frequencies included harmonic pairs, the CCA-based methods performed worse than the CNN-based methods.

**Figure 5:**
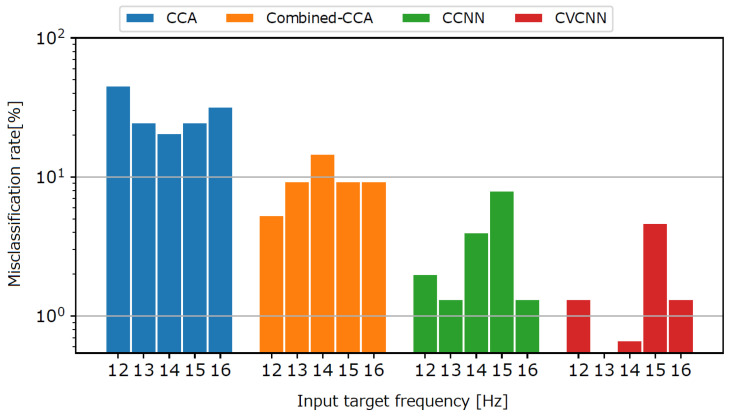
Misclassification rate for another target of harmonic pair.

To show the classification performance for the harmonic pairs, we show the misclassification rate for another target harmonic pair in Figure 5 for the 1000 ms data length. For example, the bar of 12 Hz represents the misclassification rate at which the 12 Hz target was misclassified as the 6 Hz target. The figure shows that the CCA-based methods showed higher misclassification rates than the CNN-based methods.

### 4.2. Open Dataset

**Figure 6:**
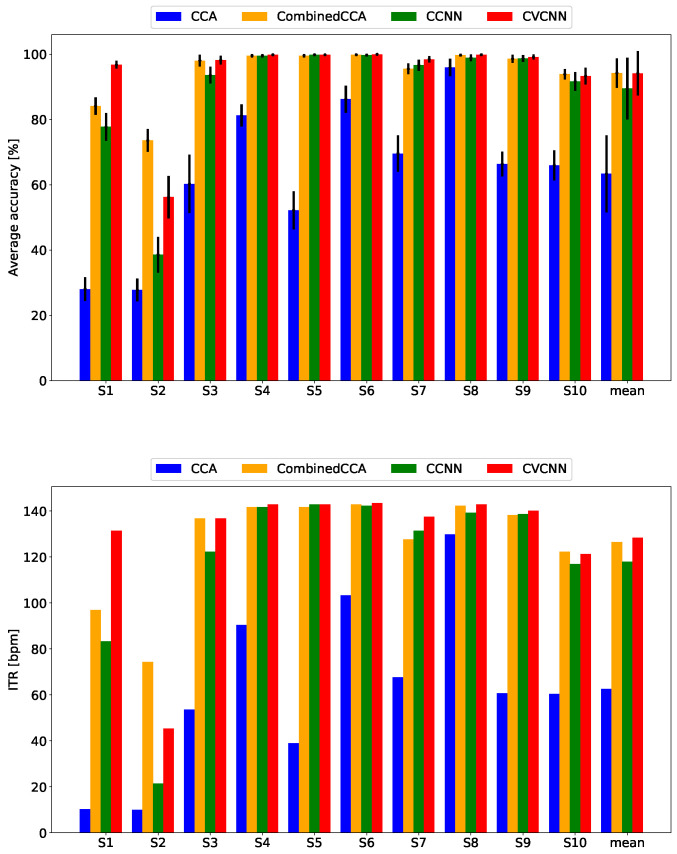
Classification accuracy and ITR for the open dataset with a data length of 1000 ms.

**Figure 7:**
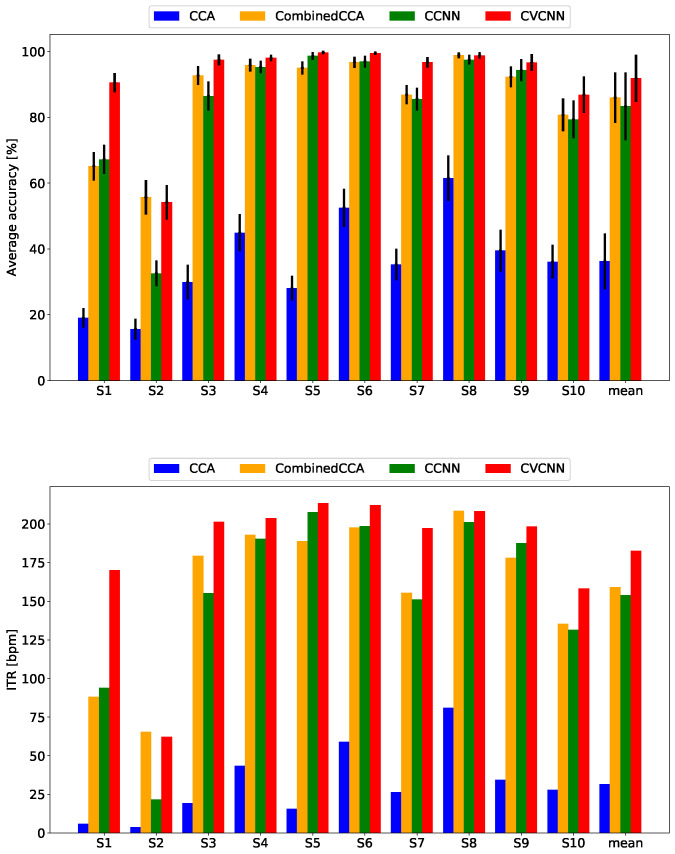
Classification accuracy and ITR for teh open dataset with a data length of 500 ms.

Figure 6 and Figure 7 show the classification accuracy and ITR for the open dataset. Although the dataset did not have harmonic flicker frequency stimuli, the proposed method exhibited the best classification accuracy and ITR for the 500 ms data length. For the 1000 ms dataset, the proposed method showed almost comparable results with the state-of-the-art method, the combined CCA, because the accuracy rates saturated to 100% for some subjects.

## 5. Conclusions

We proposed a complex valued convolutional neural network (CVCNN) structure and absolute activation function for the classification of the SSVEP-based BCI. The SSVEP-based BCI is often realized by an LCD display; however, it has a limitation of the stimulation frequency due to the refresh rate. Moreover, the conventional CCA-based SSVEP classification methods (e.g., [23,27]) have the disadvantage that it is difficult to discriminate harmonic frequency stimuli (e.g., 6 Hz and 12 Hz). This problem hinders performance improvement of the SSVEP-based BCI in terms of the number of available commands and ITR. The proposed method overcomes this problem and outperforms state-of-the-art SSVEP classification methods by using feature extraction in the frequency domain of complex valued networks and the representation learning of convolutional neural networks.

Our original data are based on a small number of participants (five subjects and four available data); therefore, further investigation is needed. Future work includes extending the proposed method for phase modulation of SSVEP applications and self-paced BCI applications.

## Figures and Tables

**Table 1:** Network structure of the proposed CNN: $N_{\mathrm{ch}}$ is the number of channels, and BN is batch normalization.

	Layer	Filters	Size	Activation
1	Input			
2	ComplexConv2D	10	$\left(N_{\mathrm{ch}},1\right)$	
3	ComplexBN			tanh(|z|)exp(jarg(z))
4	Dropout			
5	ComplexConv2D	10	(1,10)	
6	Flatten			
7	BN			ReLU(ℜ(z))+jReLU(ℑ(z))
8	Dropout			
9	ComplexDense			|z|
10	Softmax			

**Table 2:** Parameter setting of neural networks.

Parameter	Original Dataset	Open Dataset
No. of epochs	250	250
Mini-batch size	30	32
Dropout rate	{0.25,0.3,…,0.5}	{0.25,0.3,…,0.6}
Learning rate	{0.0005,0.001,0.005,0.01}	{0.001,0.005,0.01}
$L_{2}$ regularization	{1e−4,1e−31e−2}	{1e−4,1e−31e−2}

## Data Availability

The open SSVEP dataset in Section 3.2 is downloaded from https://github.com/mnakanishi/12JFPM_SSVEP (accessed on 1 June 2021).
